# A new era for optical-coupled scanning probe microscopy

**DOI:** 10.1093/nsr/nwag252

**Published:** 2026-05-07

**Authors:** Lifeng Chi

**Affiliations:** State Key Laboratory of Bioinspired Interfacial Materials Science, Institute of Functional Nano & Soft Materials (FUNSOM), Soochow University, China

Scanning probe microscopy (SPM), especially when coupled with optics, has emerged as a transformative tool for characterizing matter, offering an unparalleled combination of atomic-scale spatial resolution and chemical sensitivity. However, to achieve extreme stability at cryogenic temperatures, the traditional approach has to use liquid helium—a resource increasingly limited by rising costs and availability. While cryogen-free (dry) systems present a more sustainable alternative, they are frequently plagued by vibrations from closed-cycle cryocoolers installed on top of the SPM chamber, which typically degrades the spatial and spectroscopic resolution, thereby preventing high-quality characterization at the ångström scale [1].

In a recent article published in *Advanced Scientific Instruments*, Ma *et al*. [2] present a next-generation cryogen-free, low-temperature optical-coupled SPM system that elegantly surmounts these barriers. The key innovation lies in a remote liquefaction scheme [3] that utilizes a closed-cycle cryocooler to liquefy helium in a separate chamber, then delivering it to the microscope via a vibration-isolated flexible transfer line. This decoupling achieves a *Z*-axis stability below 1 pm and a base temperature of 2.78 K with fluctuations within ±1 mK, while the tunneling–current noise (∼20 fA/Hz^½^) remains comparable to conventional liquid-helium wet systems. Moreover, this system enables uninterrupted, month-duration operation and continuous data acquisition, thereby setting the cornerstone for time-consuming multimodal characterizations.

**Figure 1.**
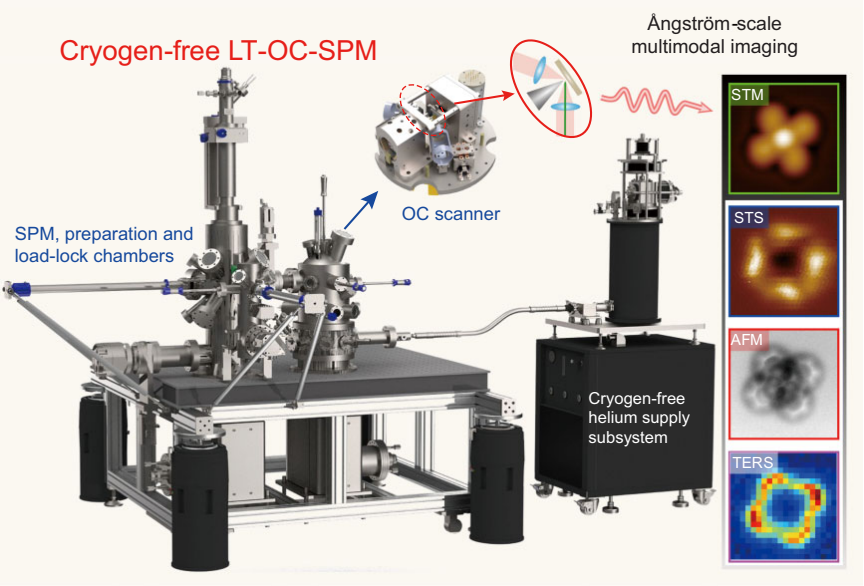
Schematic drawings of the novel cryogen-free low-temperature (LT) optical-coupled (OC) SPM system and multimodal (STM, STS, nc-AFM, TERS) characterization of AgPc/Ag(110).

By incorporating a piezo-driven high-numerical-aperture (high-NA) lens for optimized optical coupling, the authors demonstrated synchronized scanning tunneling microscopy/spectroscopy (STM/STS), non-contact atomic force microscopy (nc-AFM) and tip-enhanced Raman spectroscopy (TERS) imaging on silver phthalocyanine (AgPc) molecules (Fig. 1). The system not only resolves molecular structures and electronic states [highest occupied molecular orbit (HOMO)/lowest unoccupied molecular orbit (LUMO)] but also maps vibrational modes with clear intramolecular contrast, achieving an ångström-scale spectroscopic resolution (0.43–0.46 nm).

In summary, Ma *et al*. [2] have shown that remote-liquefaction optical-coupled SPM can provide the extreme stability required for routine single-molecule multimodal imaging without the cost of liquid helium. By synergizing multi-functional integration with ‘dry’ refrigeration, this development provides a robust foundation for long-duration characterization, marking a significant step toward sustainable atomic-scale manufacturing and unambiguous chemical identification under extreme conditions.
